# Research Progress on Antibiotic Treatment by NZVI-Based Water Treatment Technologies

**DOI:** 10.3390/toxics14080675

**Published:** 2026-07-30

**Authors:** Jun Luo, Tianhao Li, Hao Meng, Yanbing Pan, Xiaoling Lei, Yu Wen, Guoming Zeng, Da Sun

**Affiliations:** 1 School of Civiland Hydraulic Engineering, Chongqing University of Science and Technology, Chongqing 401331, Chinashuichexin@outlook.com (T.L.); 17783904908@163.com (H.M.); 13618385355@163.com (Y.P.); 2025203002@cqust.edu.cn (Y.W.); 2 Chongqing Academy of Science and Technology, Chongqing 401123, China; ellenlei2024@163.com; 3 School of Civil Engineering, Chongqing Jiaotong University, Chongqing 400074, China; 4 Zhejiang Provincial Key Laboratory of Water Ecological Environment Treatment and Resource Protection, College of Life and Environmental Science, Wenzhou University, Wenzhou 325035, China; 5 Institute of Life Sciences and Biomedical Collaborative Innovation Center of Zhejiang Province, Wenzhou University, Wenzhou 325035, China

**Keywords:** nZVI, antibiotics, modified materials, water treatment, fenton-like oxidation

## Abstract

Antibiotic contamination has become an emerging challenge in water treatment and environmental protection due to the increasing discharge of antibiotics and their transformation products, which exhibit ecological toxicity and resistance to degradation. Conventional treatment technologies often show limitations in antibiotic removal, including insufficient efficiency, high operational costs, and potential secondary pollution. Nanoscale zero-valent iron (nZVI) has attracted extensive attention as a promising remediation material for antibiotic wastewater treatment owing to its large specific surface area, strong reducing capability, and excellent adsorption properties. However, the practical application of nZVI is still restricted by particle aggregation and surface passivation, which reduce its reactivity, stability, and long-term performance. This review provides a comprehensive overview of recent advances in nZVI-based materials for antibiotic removal, with emphasis on preparation strategies, surface modification approaches, removal mechanisms, and integrated treatment technologies. The performance of pristine nZVI and modified nZVI-based systems in antibiotic degradation and removal is systematically discussed. Furthermore, current challenges, including material deactivation, iron leaching, environmental risks, and scale-up limitations, are critically evaluated, and future perspectives toward efficient, stable, and sustainable antibiotic wastewater treatment are proposed.

## 1. Introduction

Antibiotics are chemical substances produced by microorganisms, plants, and animals during growth and metabolism, and can interfere with the normal physiological functions of other living cells [1,2]. Driven by increasing demands in healthcare, aquaculture, and agriculture, the production and use of antibiotics have expanded rapidly, with β-lactams, quinolones, and tetracyclines being the major categories, as summarized in Table 1 [3,4]. In China, the development of medical infrastructure and the tiered diagnosis and treatment system have led to a continuous increase in medical wastewater discharge. According to the China Health and Wellness Statistical Yearbook and China Environmental Statistical Annual Report, the total amount of medical wastewater generated in China has risen from approximately 2.8 million m^3^/day in 2010 to over 3.5 million m^3^/day in 2023, with an average annual growth rate of about 2–3%. In parallel, agricultural antibiotic wastewater has also shown a persistent upward trend. This type of wastewater mainly originates from livestock and poultry manure, aquaculture effluents, and field runoff. China is the world’s largest producer and consumer of antibiotics; a landmark survey reported that total antibiotic usage in China was approximately 162,000 tons in 2013, of which about 52% (84,000 tons) was veterinary antibiotics used in animal husbandry. Despite regulatory control measures, the total veterinary antibiotic usage remained above 40,000 tons per year in 2020–2022, with the aquaculture sector showing an annual increase of 3–5%. It is estimated that 30–90% of administered veterinary antibiotics are excreted as parent compounds or active metabolites and eventually enter soil and water bodies through manure application, direct discharge, or surface runoff [5,6]. Wastewater from large hospitals is usually collected and discharged centrally, whereas that from primary clinics is often released in a dispersed manner, eventually entering municipal sewer systems or natural water bodies. In addition, antibiotic residues from agricultural use can persist in soil and migrate into aquatic environments through rainfall or irrigation. In livestock farming, veterinary antibiotics such as tetracycline, ceftiofur, and enrofloxacin are not fully absorbed or degraded by animals, and approximately 40% of the administered antibiotics may enter sewer systems, water bodies, or agricultural fields through livestock wastewater and manure reuse [7,8]. The continuous release of antibiotics poses chemical toxicity risks and may promote the spread of antibiotic resistance genes, extending pollution concerns to ecological genetics [9]. Even at trace levels, antibiotics can disrupt aquatic endocrine systems, disturb food-web structures, and induce biomagnification through bioaccumulation and food-chain transfer [10,11,12,13]. Therefore, antibiotic pollution has become an emerging environmental issue that urgently requires attention, and its efficient removal and risk control are of great significance for protecting aquatic ecosystems and public health. Table 1 lists common antibiotics currently studied in antibiotic pollutant removal research, along with the chemical structures of representative drugs that are widely investigated.

Conventional treatment technologies, including biological processes, adsorption, membrane separation, coagulation-flocculation, and chemical oxidation, have been widely used for antibiotic wastewater treatment [15,16,17]. However, these methods still have limitations when treating antibiotics with stable structures and high ecological risks. In China, biological methods (especially activated sludge processes) remain widely applied for hospital and pharmaceutical wastewater treatment due to their low cost and ease of operation. However, conventional biological processes generally exhibit limited efficiency toward refractory pharmaceutical pollutants. Therefore, advanced oxidation processes (AOPs, such as ozonation, Fenton-like reactions, and persulfate-based oxidation) and membrane filtration technologies have attracted increasing attention worldwide for the deep treatment of antibiotic-containing wastewater. Physical methods, such as adsorption and membrane separation, can remove antibiotics from water through enrichment or interception, but they mainly transfer pollutants to adsorbents, sludge, or concentrated streams rather than achieving complete degradation, and may suffer from adsorbent saturation, membrane fouling, and high regeneration or disposal costs [16,17]. Therefore, conventional single treatment processes are often insufficient for the efficient and safe removal of complex antibiotic pollutants, highlighting the need for new treatment strategies with simultaneous adsorption, transformation, and degradation capabilities [18]. Chemical oxidation processes, including ozonation, Fenton-like oxidation, and photocatalysis, can promote antibiotic transformation and degradation, but their performance is often affected by pH, water matrix, oxidant dosage, and energy input, and incomplete oxidation may generate transformation products with uncertain toxicity [19].

As conventional treatment technologies face limitations in the efficient removal of non-degradable antibiotics, nano-zero-valent iron (nZVI) has emerged as a promising material for antibiotic wastewater treatment because of its high surface reactivity, strong reducing ability, and adsorption capacity [20,21]. nZVI refers to zero-valent iron particles with diameters ranging from 1 to 100 nm. Due to its extremely small size, nZVI possesses a huge specific surface area (up to >35 m^2^/g) and extremely high surface energy, which endow it with a strong reducing capability far superior to that of ordinary iron powder (standard electrode potential E^0^(Fe^2+^/Fe) = −0.44 V). In addition, its unique core–shell structure provides excellent electron transfer capacity and active surface chemical reactivity [22,23]. These properties allow nZVI to remove antibiotics through multiple pathways, including adsorption enrichment, reductive transformation, and Fenton-like oxidation processes, where Fe^2+^ reacts with hydrogen peroxide to generate hydroxyl radicals that oxidize antibiotics. For antibiotics with stable aromatic rings, heteroatoms, or halogenated groups, such as tetracyclines, quinolones, and sulfonamides, nZVI-based systems can promote molecular structure transformation and enhance subsequent degradation [24,25,26,27,28]. However, pristine nZVI still suffers from particle aggregation, surface passivation, and uncertain environmental fate, which limit its long-term reactivity and practical application. Therefore, modification strategies such as surface coating, metal loading, support immobilization, and coupling with oxidation or biological processes have been widely explored to improve the stability, reactivity, and applicability of nZVI-based materials [29,30]. For example, Yan et al. used biochar-loaded nZVI to activate persulfate for tetracycline degradation [31], while Song et al. constructed a polystyrene anion-exchange resin-confined nZVI composite for ciprofloxacin removal [32]. In addition, resin-supported zero-valent iron composites have also been reported for sulfamethoxazole removal, indicating the applicability of nZVI-based materials to different classes of antibiotics [33].

Over the past decade, nZVI-based materials have attracted increasing attention in antibiotic wastewater treatment due to their strong reduction ability, high surface reactivity, and versatile adsorption–catalysis functions. Most existing studies have focused on the removal performance of specific nZVI systems or the optimization of individual modification methods, while systematic summaries addressing the preparation strategies, modification mechanisms, and coupled treatment applications of nZVI-based materials for antibiotic removal remain limited. Therefore, a comprehensive review of recent advances in nZVI-based water treatment technologies is important for understanding the relationship between material structure, reaction activity, and pollutant removal performance.

Several reviews have recently summarized the application of nZVI-based materials for antibiotic remediation and environmental pollutant removal [34]. provided an early overview of the applications of pristine and modified nZVI materials for antibiotic removal, mainly focusing on material categories, interaction mechanisms, and influencing factors. More recent reviews further discussed the effects of operational parameters, antibiotic resistance genes, and modification strategies on the performance of nZVI-based systems [20,29]. In addition, broader reviews have summarized the environmental remediation applications of nZVI based on its reductive properties and reaction characteristics [35]. However, previous reviews mainly emphasized material classification, preparation strategies, or overall removal efficiency, while the intrinsic relationship among antibiotic molecular structures, nZVI surface characteristics, and specific transformation pathways remains insufficiently understood.

In this context, this review provides a comprehensive overview of recent advances in nZVI-based materials for antibiotic wastewater treatment, with particular emphasis on preparation strategies, surface modification, material properties, and their effects on antibiotic removal mechanisms. Unlike previous reviews that mainly focused on nZVI preparation methods, modification approaches, or general removal performance, this review systematically discusses how different antibiotic structures influence their interactions with nZVI-based materials and determine the corresponding removal pathways. The roles of pristine nZVI, modified nZVI, supported nZVI, and nZVI-coupled systems are discussed from the perspectives of adsorption enrichment, electron transfer, reductive transformation, Fenton-like oxidation, and synergistic degradation processes. Furthermore, the effects of different modification strategies on surface characteristics, catalytic activity, stability, and practical application potential are critically evaluated. Finally, the challenges associated with particle aggregation, surface passivation, iron leaching, transformation products, environmental risks, and scale-up application are discussed. This review aims to provide a comprehensive understanding of the development of nZVI-based materials for antibiotic removal and offer useful guidance for the rational design and practical application of efficient wastewater treatment technologies.

## 2. Preparation and Modification of NZVI

### 2.1. Main Preparation Methods

Various preparation methods are currently available, with liquid-phase reduction, vapor deposition, solid-phase grinding, and microemulsion methods being the mainstream approaches. The characteristics of different nZVI preparation methods are compared in Table 2.

The liquid-phase reduction method features simple operation and easily controllable costs, making it dominant in both laboratory research and industrial production [36]. In this method, a strong reducing agent such as sodium borohydride rapidly reduces ferrous or ferric ions into zero-valent iron atoms in the liquid phase, resulting in a vigorous reaction. The reactant concentration, pH, and addition rate are key factors controlling crystal nucleus growth and agglomeration. However, residual byproducts from sodium borohydride may form a passivation layer on the surface, hindering electron transfer. The use of surfactants or polymer supports can improve dispersibility, but the instability of the material in aqueous environments limits its long-term application (see Equation (1)). Additionally, Kharagpur et al. used a novel hydrazine to prepare nZVI and compared it with the traditional sodium borohydride method; their results showed that the new hydrazine reduction route produced smaller, relatively uniform, narrowly distributed, and spherical nZVI particles [37]. Ruiz-Torres et al. used ethylene glycol as a surface functionalization agent to prepare air-stable nZVI without inert conditions, and their results showed that the EG functionalization route enabled synthesis under ambient atmosphere, produced smaller and more narrowly distributed spherical nZVI particles with enhanced colloidal dispersibility and effective protection against oxidation, and maintained high contaminant reduction reactivity comparable to that of conventionally prepared nZVI; however, EG functionalization did not fundamentally alter the magnetic dipole-driven chain-like aggregation behavior of the particles and the mono-dentate coordination still permitted a minor degree of surface oxidation over time [38].

(1) $$Fe^{2+}+2BH_{4}^{-}+6H_{2}O\to Fe^{0}↓+2B(\mathrm{OH}{)}_{3}+7H_{2}↑$$

Gas-phase reduction method typically involves high-temperature pyrolysis of a gaseous iron source followed by reduction with hydrogen (typically in the range of 300–600 °C) [39]. The nZVI prepared by this method exhibits high purity, good crystallinity, few surface defects, and strong oxidation resistance. However, it suffers from high energy consumption and requires a vacuum or inert atmosphere, which poses obstacles to its engineering application. Particles prepared via the vapor phase method have insufficient surface roughness and functional groups, resulting in poor wettability and affinity in medical wastewater, thereby reducing the probability of collision with pollutant molecules. Therefore, this method is mostly used in fundamental research that demands high material purity, such as for catalyst supports, while its application in water treatment is costly and yields limited effectiveness (see Equation (2)) [40].

(2) $$Fe_{2}O_{3}\left(s\right)+3H_{2}\left(g\right)\to 2Fe^{0}\left(s\right)+3H_{2}O\left(g\right)$$

The solid-phase reduction method involves mixing an iron salt and a reducing agent via high-energy ball milling, using mechanical energy to initiate a solid-state chemical reaction without the need for a solvent, thereby avoiding the secondary wastewater issue associated with liquid-phase methods [40]. The mechanical force can refine the grains and generate high-density dislocations and defects through lattice distortion, creating highly active reactive sites. However, the localized high temperatures during ball milling may cause sintering and cold welding of iron particles, resulting in a broad particle size distribution. Controlling the milling speed, duration, and ball-to-powder ratio is key to balancing grain refinement and agglomeration. This method struggles to produce nanoparticles with uniform morphology and extremely small particle sizes; the resulting product is often a mixture of micron- and nano-scale particles, making it less suitable for fine water treatment applications (see Equation (3)).

(3) $$Fe_{2}O_{3}\left(s\right)+3\mathrm{Mg}\left(s\right)\to 2Fe^{0}\left(s\right)+3\mathrm{MgO}\left(s\right)$$

The microemulsion method is based on colloid chemistry, using surfactants to form water-in-oil (W/O) nanoreactors, in which the iron salt and reducing agent react within tiny “water pools” [41]. The core reaction equation is the same as that of the liquid-phase reduction method. This method controls the boundaries of grain growth through spatial confinement, enabling the production of nanoparticles with uniform particle size, good dispersibility, and controllable dimensions. The “micro-zone isolation effect” prevents particle agglomeration. However, this method suffers from low yield and high reagent consumption, which increases costs, and it may also introduce new organic pollutants, causing secondary contamination.

**Table 2:** Comparison of the main preparation methods and properties of nZVI.

Preparation Method	Reaction Principle	Technical Features	Product Characteristics	Advantages and Disadvantages	References
Liquid-phase reduction method	Fe^2+^/Fe^3+^ reacts with a reducing agent such as NaBH_4_ to form Fe^0^	Simple operation and mild reaction conditions	Small particle size (1–100 nm), good dispersibility	Advantages: Low cost, easy to scale; Disadvantages: Prone to agglomeration, requires inert gas protection	[36]
Gas-phase reduction method	Fe precursors decompose/reduce at high temperatures, and gas-phase atoms deposit into nanoparticles	The purity of the product is high and the particle size is controllable	High crystallinity and strong surface activity	Advantages: Stable product quality; Disadvantages: Complex equipment, high energy consumption	[40]
Solid-state reduction method	The micron-sized Fe powder is crushed to the nanometer level by mechanical force	The process is simple and the raw materials are readily available	It has a wide particle size distribution and many surface defects	Advantages: Easy to operate and low cost; Disadvantages: Prone to introducing impurities, severe agglomeration	[40]
Microemulsion method	Fe ions are deposited as nanoparticles by cathodic electroreduction	Particle size is controllable and morphology is uniform	Clean surface, high purity	Advantages: Controllable product morphology; Disadvantages: High energy consumption, low production efficiency	[41]

### 2.2. Modification Strategies

Surface coating involves encapsulating nZVI with a layer of organic or inorganic material, which inhibits particle agglomeration through steric hindrance or electrostatic repulsion, while simultaneously shielding the particles from oxygen and moisture to delay surface passivation [42]. Commonly used coating materials include natural polymers, synthetic polymers, and inorganic gels.

Polyethylene glycol (PEG), as a commonly studied stabilizer, was employed by Zhao et al., who used PEG-4000 to modify and support nZVI on zeolite to prepare a polyethylene glycol-stabilized nano-zero-valent iron composite loaded on zeolite (PZ-nZVI). The resulting nZVI particles exhibited uniform dispersion with significantly reduced agglomeration, achieving adsorption capacities of 54.67 and 48.88 mg/g for norfloxacin and ofloxacin, respectively [43]. Starch, an inexpensive and biodegradable natural polymer, has also been utilized for nZVI stabilization. Almojil and Almohana synthesized starch-stabilized nZVI for the degradation of flutamide in an ultrasound/persulfate system. After ten cycles, the catalyst exhibited only a 6.22% loss in activity with low iron leaching, demonstrating excellent stability [44]. Although the experiments were limited to 10 cycles, it can be expected that if the number of cycles were increased (e.g., to 20 or 30 cycles), the activity might gradually decline due to cumulative iron leaching and surface fouling [44]. Inorganic gel coatings, on the other hand, form a dense protective layer to inhibit nZVI oxidation. Wang et al. designed aluminum hydroxide gel-coated nZVI (AHG@NZVI), which not only enhanced particle dispersion but also promoted pollutant adsorption onto the nZVI surface by adsorbing Fe^2+^/Fe^3+^, and achieved synergistic tetracycline removal through flocculation, reaching a removal efficiency of 98.1% within 60 min [45].

Loading nZVI onto porous or high-surface-area supports enables physical isolation of nZVI particles while simultaneously concentrating pollutants through adsorption, thereby reducing the distance between contaminants and active sites. Additionally, the conductivity or catalytic activity of the support itself can participate in electron transfer, enhancing overall reaction efficiency [42,46,47].

Activated carbon, as the most widely used support material, was employed by Yun et al., who loaded nZVI onto powdered activated carbon (PAC) to prepare nZVI@PAC for the activation of H_2_O_2_ to degrade ofloxacin, achieving a removal efficiency of 94.7% [48]. Li et al. utilized alkali-modified biochar-supported nZVI (MBC@nZVI) to activate persulfate for naphthalene degradation, achieving 96% removal within 60 min, with the material maintaining high activity across a pH range of 3–9 [49]. In addition to naphthalene, several other studies have demonstrated the effectiveness of activated carbon- or biochar-supported nZVI for the removal of various polycyclic aromatic hydrocarbons (PAHs). For instance, composites have shown high removal efficiencies for phenanthrene, pyrene, and fluoranthene, primarily through combined adsorption and catalytic oxidation mechanisms [50]. Zhao Ruohan et al. prepared a calcium alginate-nZVI-rice straw biochar composite (CANRC) via a calcium alginate-entrapment and liquid-phase reduction method for the removal of Pb^2+^, Zn^2+^, and Cd^2+^ in water, achieving high removal efficiencies with maximum adsorption capacities of 345.5 mg/g for Pb^2+^, 182.3 mg/g for Zn^2+^, and 156.8 mg/g for Cd^2+^ within 120 min [36]. Yun et al. also prepared an nZVI@PAC composite using powdered activated carbon (PAC) as a support via an ultrasound-assisted chemical co-precipitation method, which was subsequently employed to activate H_2_O_2_ for ofloxacin degradation [48].

Zeolite molecular sieves, with their well-defined pore structures and ion-exchange capacity, are also commonly used as supports. Zhao et al. loaded PEG-stabilized nZVI onto zeolite, achieving efficient adsorption of two fluoroquinolone antibiotics [28]. Wang et al. developed chitosan-modified ZSM-5 molecular sieve-supported nZVI (NZVI@C-ZSM), whereby the abundant amino and carboxyl groups on the surface anchored the iron particles. Synthesized without the need for nitrogen protection, this material achieved a tetracycline degradation efficiency of 97.72% within 60 min, with significantly lower iron leaching compared to the unmodified material [47,51]. Natural materials have also been explored as supports. Moreno-Bárcenas et al. prepared a jute fiber-supported nZVI/GO hybrid (J-nZVI/GO) using natural jute fibers as a support and investigated its arsenic removal performance in dynamic flow filtration systems [52].

Metal doping involves introducing a second metal (e.g., Cu, Ni, Pd, Bi, Co) into nZVI to form bimetallic nanoparticles, thereby creating a micro-galvanic cell that significantly alters the surface electronic properties, enhancing reactivity and selectivity. The doped metal typically possesses a higher hydrogen overpotential or catalytic activity, acting as an electron transfer mediator that facilitates the generation of atomic hydrogen or directly participates in activator oxidation. Su et al. synthesized Bi-doped nZVI (Bi/nZVI) and employed it to activate molecular oxygen in a citrate buffer system for the degradation of sulfonamide antibiotics. The introduction of Bi accelerated the regeneration of Fe^2+^ and the generation of hydroxyl radicals (·OH), achieving complete removal of sulfamethoxazole within 2 h [53]. Nandana B et al. prepared nZVI/Cu-PVDF hybrid membranes. The introduction of Cu accelerated the regeneration of Fe^2+^ and the generation of ·OH. Within 180 min, the removal rate of CIP (ciprofloxacin) reached 94%, and that of TET (tetracycline) reached 80% [15]. Weng et al. prepared bentonite-supported Fe/Ni bimetallic nanoparticles (B-Fe/Ni), which simultaneously reduced and degraded β-lactam antibiotics under anaerobic conditions. The introduction of Ni promoted electron transfer and hydrogen radical generation. Within 60 min, the removal rates of amoxicillin, ampicillin and penicillin reached 94.6%, 80.6% and 53.7%, respectively [54].

### 2.3. Mechanism of nZVI and Modified nZVI

The mechanism of individual nZVI in water treatment can be broadly categorized into reductive degradation and oxidative degradation. Reductive degradation is primarily achieved through electron transfer, such as the reductive detoxification of halogenated organic compounds or nitroaromatic antibiotics (see Equation (4)).

(4) $$Fe^{0}+\mathrm{RCl}+H^{+}\to Fe^{2+}+\mathrm{RH}+Cl^{-}$$

For support-loaded nZVI, the underlying mechanisms mainly involve adsorption-catalysis synergy, accelerated electron transfer and reactive species generation, regulation of iron cycling and catalytic durability, and modulation of radical generation pathways.

Supports such as activated carbon and biochar concentrate pollutants through adsorption, thereby increasing local concentrations and accelerating catalytic reactions. Conductive supports can act as electron shuttles, facilitating electron transfer from nZVI to pollutants or oxidants. Deng et al. incorporated nZVI into a g-C_3_N_4_ composite for the degradation of ciprofloxacin in a dielectric barrier discharge plasma system, where g-C_3_N_4_ served as an electron transfer platform, significantly enhancing the generation efficiency of reactive species and achieving a removal efficiency of 94.3% within 10 min [55]. Density functional theory (DFT) calculations revealed that Fe atoms in the nZVI/g-C_3_N_4_ composite tend to lose electrons, promoting the dissociation of adsorbed water to generate ·OH (see Equation (5)). Additionally, supports can prolong the service life of the system by promoting the cyclic reaction between Fe^3+^ and Fe^2+^ ions [56]. The reduction reaction of Fe^3+^ can be expressed as Equation (6). The mechanism of metal doping mainly manifests in three aspects: constructing electron transfer pathways, modulating radical contribution ratios, and expanding the applicable pH range.

(5) $$H_{2}O_{\mathrm{ads}}\;\to \cdot OH_{\mathrm{ads}}+H^{+}+e^{-}$$

(6) $$2Fe^{3+}+Fe^{0}\to 3Fe^{2+}$$

Through metal doping, the secondary metal often serves as an electron transfer mediator, accelerating electron transfer from the core to the surface of nZVI. Taking Fe/Cu bimetallic particles as an example (see Equations (7) and (8)). The presence of Cu accelerates the regeneration of Fe^2+^ ions, thereby sustaining the continuous generation of radicals in the system [42].

(7) $$Cu^{0}+S_{2}O_{8}^{2-}\to Cu^{2+}+SO_{4}^{2-}+SO_{4}^{-}$$

(8) $$Cu^{2+}+Fe^{0}\;\to Cu^{0}+Fe^{2+}$$

Furthermore, different metal dopants can alter the contribution of various radicals. Bai et al. investigated the contribution of radicals in an ultrasound/S-nZVI/peracetic acid system under different pH conditions, finding that ·OH contributed more significantly under acidic conditions, while the contributions of RO and Fe(IV) increased with rising pH [57].

Metal doping can also enhance the activity of nZVI over a wide pH range. Su et al. developed Bi-doped nZVI (Bi/nZVI) in a citrate buffer system, where the introduction of Bi allowed the material to maintain high activity across a broad pH range [53].

### 2.4. Sulfidation Modification and Fenton-like Systems

Sulfidation involves the formation of an iron sulfide (e.g., FeS, FeS_2_) layer on the surface of nZVI, thereby altering its surface hydrophobicity and electron selectivity [42,49,57]. Sulfidation modification has gained considerable popularity in recent years; according to recent literature surveys, sulfidation is among the most frequently reported modification strategies for nZVI, accounting for roughly 25–30% of all surface engineering studies aimed at improving electron selectivity [58]. The sulfidation layer not only inhibits excessive corrosion of nZVI but also preferentially directs electrons toward target pollutants rather than water or dissolved oxygen, thereby improving electron utilization efficiency [59]. The sulfidation process typically involves treating nZVI with sulfides such as Na_2_S (see Equation (9)). Fenton-like systems, on the other hand, refer to processes in which iron-based materials activate oxidants such as hydrogen peroxide (H_2_O_2_), persulfate (PS, including peroxymonosulfate (PMS) and peroxydisulfate (PDS)), or peracetic acid (PAA) to generate highly reactive radicals (e.g., ·OH, SO_4_^⋅−^, organic radicals), thereby enabling efficient mineralization and degradation of organic pollutants [25,60].

(9) $$Fe^{0}+S^{2-}+2H_{2}O\to \mathrm{FeS}+H_{2}↑+2OH^{-}$$

Sulfidation reactions are typically carried out in aqueous solutions, where a sulfur source reacts with the nZVI surface to form a sulfidation layer, accompanied by the release of hydrogen gas (see Equation (9)). The resulting sulfidated nZVI (S-nZVI) possesses a core–shell structure, with an inner layer of Fe^0^ and an outer layer composed of iron sulfides and a small amount of iron oxides [8]. The sulfidation layer exhibits hydrophobic properties, which suppress side reactions between nZVI and water (hydrogen evolution), thereby directing more electrons toward the target pollutants and improving electron utilization efficiency. Zou et al. prepared biochar from rice straw and loaded it with sulfidated nZVI (S-nZVI/BC) to activate persulfate for the degradation of ten different sulfonamide antibiotics, achieving a removal efficiency of up to 98.3% [61]. Li et al. found that in the MBC@S-nZVI system for naphthalene degradation via persulfate activation, the presence of the sulfidation layer enabled preferential electron transfer to pollutants rather than to water or dissolved oxygen, resulting in significantly enhanced degradation efficiency [49].

The sulfidation layer also acts as an electron conductor, promoting the Fe^3+^/Fe^2+^ redox cycle. Liang et al. demonstrated that the sulfidated S-nZVI/BC exhibited significantly better tetracycline degradation performance compared to non-sulfidated nZVI/BC. In their electron spin resonance (ESR) tests, the S-nZVI/BC system generated more OH and O_2_^−^ radicals, which was attributed to the accelerated Fe^2+^/Fe^3+^ cycling facilitated by FeS (see Equation (10)). FeS has a narrower band gap and higher electrical conductivity than iron oxides, enabling more efficient electron transfer from the Fe^0^ core to the surface-bound oxidants, thus maintaining a higher steady-state concentration of reactive radicals [62].

(10) $$2Fe^{3+}+\mathrm{FeS}\to 3Fe^{2+}+S^{2-}$$

In practice, the actual sulfidation efficacy is closely related to the S/Fe molar ratio in the system. A ratio that is too low results in an incomplete sulfidation layer that cannot effectively protect the core, while an excessively high ratio may lead to the formation of excessive FeS_2_, which can block active sites and consume excessive Fe^0^ [42,49]. In the S-nZVI/PAA system investigated by Bai et al., the optimal S/Fe molar ratio was found to be 1:20, achieving an enrofloxacin removal efficiency of 87.13%; further increasing the S/Fe ratio to 1:5 resulted in a decrease in removal efficiency to 76.57% [57,63].

In Fenton-like reactions where nZVI activates H_2_O_2_, nZVI corrodes in water to release Fe^2+^ ions, which subsequently react with H_2_O_2_ to generate ·OH (see Equations (11) and (12)). Liang et al. found that the nZVI/H_2_O_2_ system achieved over 90% removal of diclofenac within 120 min at pH 5, with a TOC removal efficiency of 85%, demonstrating its excellent mineralization capability [64].

(11) $$Fe^{0}+2H^{+}\to Fe^{2+}+H_{2}$$

(12) $$Fe^{2+}+H_{2}O_{2}\to Fe^{3+}+\cdot \mathrm{OH}+OH^{-}$$

The activation of persulfate by nZVI generates SO_4_^⋅−^, under alkaline conditions, SO_4_^⋅−^ can be converted to ·OH (see Equations (13)–(16)) [49]. Peracetic acid (PAA) activated by nZVI can generate organic radicals (such as CH_3_C(O)O· and CH_3_C(O)OO·) along with ·OH [49]. In addition to radical pathways, non-radical pathways may also be involved in nZVI-based Fenton-like systems, such as the participation of singlet oxygen (^1^O_2_) (see Equations (17)–(22)) [49,53]. Su et al. demonstrated that in a Bi/nZVI/citrate system activating molecular oxygen, ^1^O_2_ contributed significantly to the degradation of sulfonamide antibiotics [53].

(13) $$Fe^{0}+S_{2}O_{8}^{2-}\to Fe^{2+}+2SO_{4}^{2-}$$

(14) $$Fe^{2+}+S_{2}O_{8}^{2-}\to Fe^{3+}+SO_{4}^{2-}+SO_{4}^{-}$$

(15) $$SO_{4}^{-}+H_{2}O\to SO_{4}^{2-}+\cdot \mathrm{OH}+H^{+}$$

(16) $$SO_{4}^{-}+OH^{-}\to SO_{4}^{2-}+\cdot \mathrm{OH}$$

(17) $$CH_{3}C\left(O\right)\mathrm{OOH}+Fe^{2+}\to CH_{3}C\left(O\right)O\cdot +Fe^{3+}+OH^{-}$$

(18) $$CH_{3}C\left(O\right)\mathrm{OOH}+Fe^{2+}\to CH_{3}C\left(O\right)\mathrm{OH}+Fe^{\mathrm{IV}}O^{2+}$$

(19) $$CH_{3}C\left(O\right)O\cdot \to \cdot CH_{3}+CO_{2}$$

(20) $$S_{2}O_{8}^{2-}+2H_{2}O\to 2SO_{4}^{2-}+HO_{2}^{-}+3H^{+}$$

(21) $$S_{2}O_{8}^{2-}+HO_{2}^{-}\to SO_{4}^{2-}+SO_{4}^{-}+\cdot O_{2}^{-}+H^{+}$$

(22) 2⋅O2−+2H+→O21+H2O2

The pH of the solution exerts a considerable influence on nZVI-based Fenton-like systems. Acidic conditions facilitate Fe^2+^ release and radical generation; however, excessively low pH (below 3) can lead to competition for electrons by H^+^ and radical quenching [65]. Luo et al. reported that the nZVI/H_2_O_2_ system achieved complete degradation of sulfadiazine within 5 min at pH 2.44, whereas the removal efficiency decreased at pH values above 7 [66]. Increasing the oxidant concentration appropriately can enhance radical yield, but an excess of oxidant can induce radical self-quenching [48]. Rahmani et al. observed that in an nZVI/PS system, the removal efficiency of ciprofloxacin increased as the PS concentration was raised from 450 mg/L to 1200 mg/L, but decreased upon further increase to 1455 mg/L [65]. Xiao et al. found that in a DTPA-enhanced nZVI/H_2_O_2_ system under neutral pH conditions, DTPA effectively prevented surface passivation of nZVI by forming soluble complexes with Fe^2+^, thereby extending the applicable pH range of the system [67].

The combination of nZVI with semiconductor photocatalysts to construct heterojunction structures enables a synergistic effect between photocatalysis and Fenton-like reactions, thereby significantly enhancing the removal efficiency of non-degradable antibiotics [68].

Wang et al. innovatively designed a double S-scheme heterojunction photocatalyst, Fe@Bi_2_MoO_6_@BiOI. In this study, the passivation layer (Fe_2_O_3_) of nZVI was strategically utilized as a semiconductor to construct a double heterojunction with Bi_2_MoO_6_ and BiOI [69]. Wu et al. designed a novel Z-scheme heterojunction catalyst, Fe^0^@CQDs-TiO_2_(b), for the degradation of metronidazole (MNZ) under visible light [68]. The introduction of CQDs promoted the generation of ·OH during dark reactions and optimized the band structure of black TiO_2_. Furthermore, the Z-scheme heterojunction constructed between the passivation layer of nZVI (Fe_2_O_3_) and CQDs-TiO_2_(b) improved the separation and migration efficiency of photogenerated carriers. In another study, Wei et al. prepared a bimetallic nanomaterial, nCo@nZVI, modified with nano-cobalt, which removes tetracycline through a synergistic “adsorption-degradation” mechanism. The material not only exhibits an adsorption capacity of 25.33 mg/g but also maintains 65.87% removal efficiency after five cycles of reuse [70]. TEM characterization revealed that nZVI/Ni and nZVI/Cu particles were uniformly dispersed on the surface of MCM-41, effectively addressing the agglomeration issues commonly associated with nZVI-based bimetallic nanoparticles.

## 3. Removal of Different Antibiotics

### 3.1. Tetracycline

Tetracycline (TC), one of the broad-spectrum antibiotics, is widely used in human medicine, animal husbandry, and aquaculture due to its strong antibacterial activity and low cost [64,71]. However, the metabolic rate of tetracycline in organisms is low, with approximately 50–80% of the administered tetracycline being excreted into the environment as parent compounds or metabolites via feces and urine [51]. Consequently, tetracycline is frequently detected in surface water, groundwater, and even drinking water [71,72]. The continuous accumulation of TC in aquatic environments may cause adverse ecological effects, including inhibition of microbial community activity, disruption of aquatic ecosystem balance, and potential toxicity to aquatic organisms. In addition, the persistent exposure of microorganisms to sub-lethal concentrations of antibiotics can accelerate the selection and dissemination of antibiotic resistance genes (ARGs), thereby reducing the effectiveness of antibiotic therapies and posing potential risks to environmental safety and human health [73,74].

The molecular structure of tetracycline (C_22_H_24_N_2_O_8_) contains multiple functional groups, including phenolic hydroxyl groups, enol groups, a dimethylamino group, and an amide group. Among these, the phenolic hydroxyl and enol groups can form stable complexes with metal ions (Fe^2+^/Fe^3+^), which is a key factor for its adsorption and removal by nZVI [28,75].

**Figure 1:**
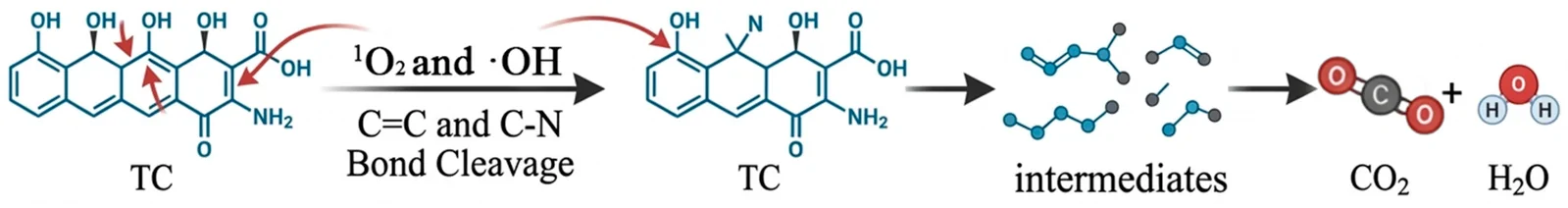
Tetracycline degradation pathway [79].

With respect to adsorption, nZVI and its oxidation products (e.g., Fe_2_O_3_, Fe_3_O_4_, FeOOH) possess abundant surface hydroxyl groups and can adsorb tetracycline through electrostatic interactions, hydrogen bonding, and surface complexation. Wang et al. found that within a pH range of 5–7, tetracycline exists as zwitterions or anions and undergoes electrostatic attraction with the positively charged surface of nZVI@G-GEL, resulting in maximum adsorption capacity. In contrast, electrostatic repulsion under strongly acidic or strongly alkaline conditions leads to a decrease in adsorption [45]. In addition to electrostatic adsorption, the phenolic hydroxyl and enol groups of tetracycline can form bidentate or multidentate complexes with iron ions on the nZVI surface. XPS analysis in the study by Wang et al. confirmed the formation of Fe–O coordination bonds between tetracycline and iron on the NZVI@C-ZSM surface (see Equations (23) and (24)) [51]. When nZVI is loaded onto carbon-based materials such as biochar or graphene, π-π stacking interactions can occur between the aromatic structures of the support and the aromatic rings of tetracycline [72,76]. Under anoxic conditions, nZVI can reduce the unsaturated functional groups in tetracycline molecules through direct electron transfer [45]. Wang et al. demonstrated that tetracycline undergoes deamination, decarbonylation, and dehydroxylation reactions in the presence of nZVI (see Equation (25) and Figure 1) [45]. In contrast, under oxic conditions, nZVI activates dissolved oxygen (DO) to generate reactive oxygen species (ROS), which subsequently oxidize tetracycline (see Equations (26)–(29)) [53].

(23) $$Fe^{2+}+\mathrm{TC}\to \mathrm{Fe}\left(\mathrm{II}\right)\text{-}\mathrm{TC}\;\mathrm{complex}$$

(24) $$Fe^{3+}+\mathrm{TC}\to \mathrm{Fe}\left(\mathrm{III}\right)\text{-}\mathrm{TC}\;\mathrm{complex}$$

(25) $$\mathrm{TC}+2e^{-}+2H^{+}\to \mathrm{TC}\left(-2H\right)+H_{2}O$$

(26) $$Fe^{0}+O_{2}+2H^{+}\to Fe^{2+}+H_{2}O_{2}$$

(27) $$Fe^{2+}+H_{2}O_{2}\to Fe^{3+}+\cdot \mathrm{OH}+OH^{-}$$

(28) $$Fe^{2+}+O_{2}\to Fe^{3+}+\cdot O_{2}^{-}$$

(29) 2⋅O2−+2H+→O21+H2O2

Wang et al. employed a glycine-modified gelatin aerogel-supported nZVI (NZVI@G-GEL) system and identified three types of reactive oxygen species (·OH, ·O_2_^−^, and ^1^O_2_) via EPR analysis, with their respective contributions to tetracycline degradation determined to be 11.22%, 65.67%, and 31.09% [77]. In Fenton-like systems, the addition of exogenous oxidants such as H_2_O_2_ and persulfate can significantly enhance radical yield [48,65,78]. Song et al. utilized a Cu-doped nZVI composite with nanocracks (nZVIC@NPS) for the simultaneous reduction of Cr(VI) and degradation of sulfamethoxazole, achieving removal efficiencies of 99.78% for Cr(VI) and 96.35% for SMX [78]. Yan et al. prepared a biochar-supported nZVI (BC-nZVI) composite via a liquid-phase reduction method and employed it to activate persulfate for tetracycline (TC) degradation. Under optimal conditions (Fe:C = 1:1, BC-nZVI dosage of 10 mg, PS concentration of 0.25 mM, pH = 3), the TC degradation efficiency reached 99.57% within 120 min [31]. Shao et al. prepared sulfurized modified nZVI (S-nZVI) and activated the degradation of tetracycline in a peracetic acid system. The sulfurization modification accelerated the conversion of Fe(III) to Fe(II) and the generation of acetyl (per) oxygen radicals [79]. Zhao et al. constructed a symbiotic system of Chlorella—endophytic bacteria—Penicillium roseopurpureum mediated by nZVI to treat nutrients and tetracycline antibiotics in aquaculture wastewater. NZVI promoted the growth of microalgae and the improvement of photosynthetic efficiency. Within 10 days, the removal rate of tetracycline reached 99.17 ± 0.52% [80].

A comparison of various nZVI-based materials for tetracycline treatment is presented in Table 3.

### 3.2. β-Lactam Antibiotics

β-lactam antibiotics mainly include penicillins (such as amoxicillin and ampicillin) and cephalosporins (such as cephalexin) [83]. The molecular structure of this class of antibiotics commonly contains a β-lactam four-membered ring, which is critical for their antibacterial activity. However, this ring also renders them resistant to natural degradation in the environment. Due to its high ring strain, the β-lactam ring is chemically reactive and prone to ring-opening reactions under acidic, alkaline, or enzymatic conditions. Taking amoxicillin (AMX) and ampicillin (AMP) as examples, the β-lactam ring and the thiazolidine ring in their molecular structures serve as the primary reactive sites [84]. Although β-lactam antibiotics are highly effective antibacterial agents, their widespread use and continuous discharge into aquatic environments may lead to the enrichment of β-lactam-resistant bacteria and disturb microbial ecosystems. In particular, the persistence of β-lactam residues can promote the selection and dissemination of microorganisms carrying β-lactamase resistance genes, thereby accelerating the spread of antibiotic resistance in the environment. Moreover, the emergence of β-lactam-resistant pathogens may compromise the clinical effectiveness of commonly used β-lactam antibiotics, resulting in treatment failure, prolonged infections, and increased challenges in human health management. In addition, transformation products generated during environmental degradation may exhibit different biological activities and should also be considered as potential environmental concerns [85].

Ghauch et al. demonstrated that nZVI can induce the ring-opening degradation of β-lactam antibiotics through reductive pathways. Under anoxic conditions, atomic hydrogen (H*) generated from the reaction of nZVI with water, or direct electron transfer, attacks the carbonyl carbon of the β-lactam ring, leading to C–N bond cleavage and the formation of the corresponding penicilloic acid. Taking amoxicillin as an example, the reductive ring-opening reaction is as in Equation (30).

(30) $$\mathrm{AMX}+2H^{*}\to \mathrm{AMX}\text{-}\mathrm{penicilloic}\;\mathrm{acid}$$

**Figure 2:**
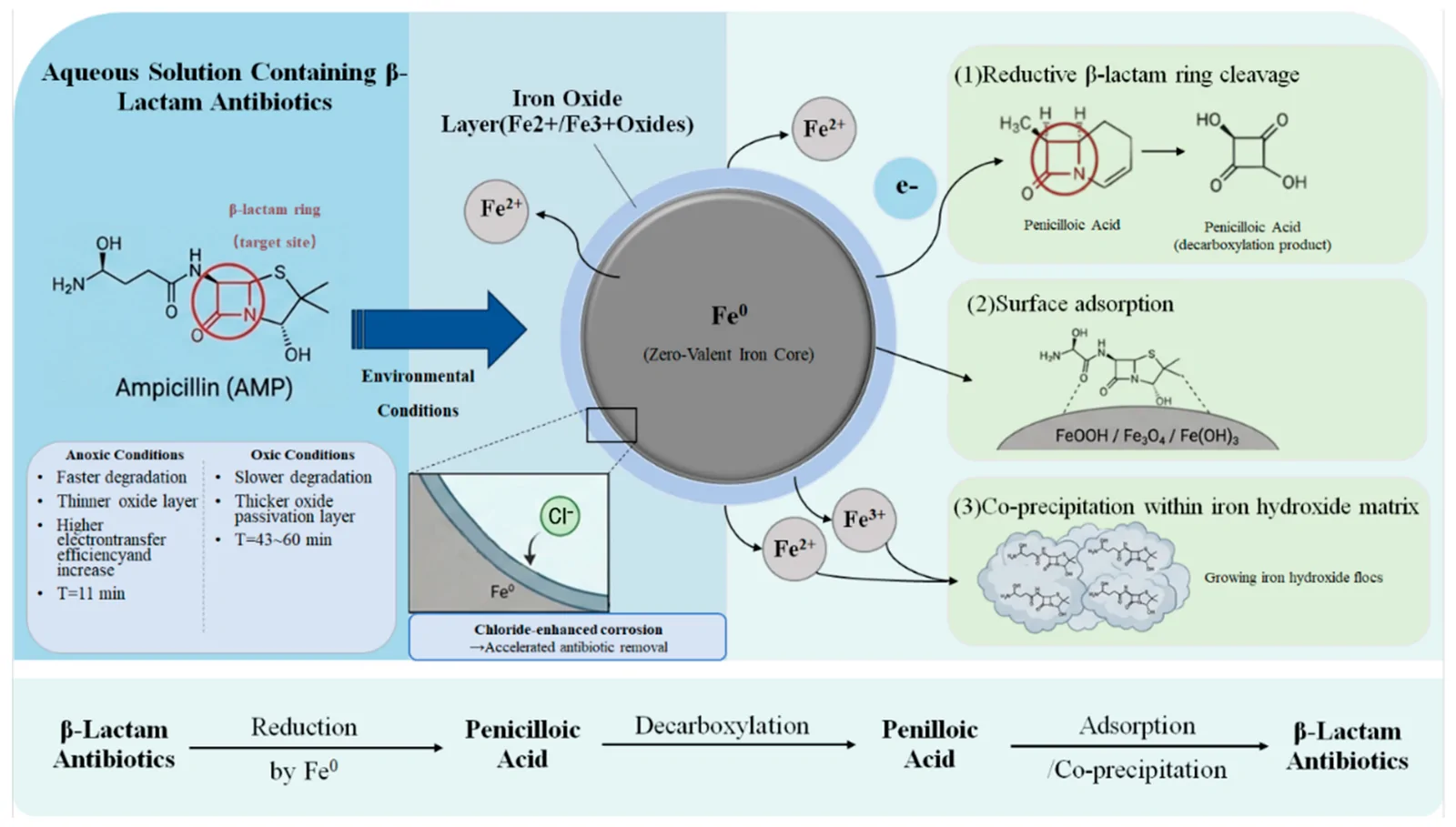
nZVI removes the β -lactam antibiotic (ampicillin) pathway [14].

Under oxic conditions, oxides and hydroxides form on the nZVI surface, enabling the adsorption of β-lactam antibiotics through surface complexation and electrostatic interactions. Additionally, the oxides can form coordination bonds with functional groups of the antibiotic molecules, facilitating adsorption and removal. Furthermore, nZVI can activate dissolved oxygen to generate H_2_O_2_, which subsequently produces hydroxyl radicals (·OH) via Fenton reactions, leading to the oxidative degradation of β-lactam antibiotics [46]. Concurrently, the generation of Fe^2+^ and Fe^3+^ ions results in the formation of hydroxide precipitates under neutral conditions, and antibiotic molecules can be entrapped and removed through this flocculation and precipitation process (see Equations (31) and (32) and Figure 2).

(31) $$Fe^{3+}+3OH^{-}\to \mathrm{Fe}(\mathrm{OH}{)}_{3}\;↓$$

(32) $$Fe(\mathrm{OH}{)}_{3}+\mathrm{AMX}\to \mathrm{Fe}(\mathrm{OH}{)}_{3}\text{-}\mathrm{AMX}\;(\mathrm{co}\text{-}\mathrm{precipitation})$$

A comparison of various nZVI-based methods for treating β-lactam antibiotics is shown in Table 4.

### 3.3. Fluoroquinolones

Fluoroquinolone antibiotics (FQs) include ciprofloxacin (CIP), norfloxacin (NOR), ofloxacin (OFL), and enrofloxacin (ENR) [43,90,91]. The molecular structure of this class of antibiotics commonly contains a quinolone ring, a piperazine ring, and a fluorine atom, contributing to their chemical stability and resistance to biodegradation [92,93].

**Figure 3:**
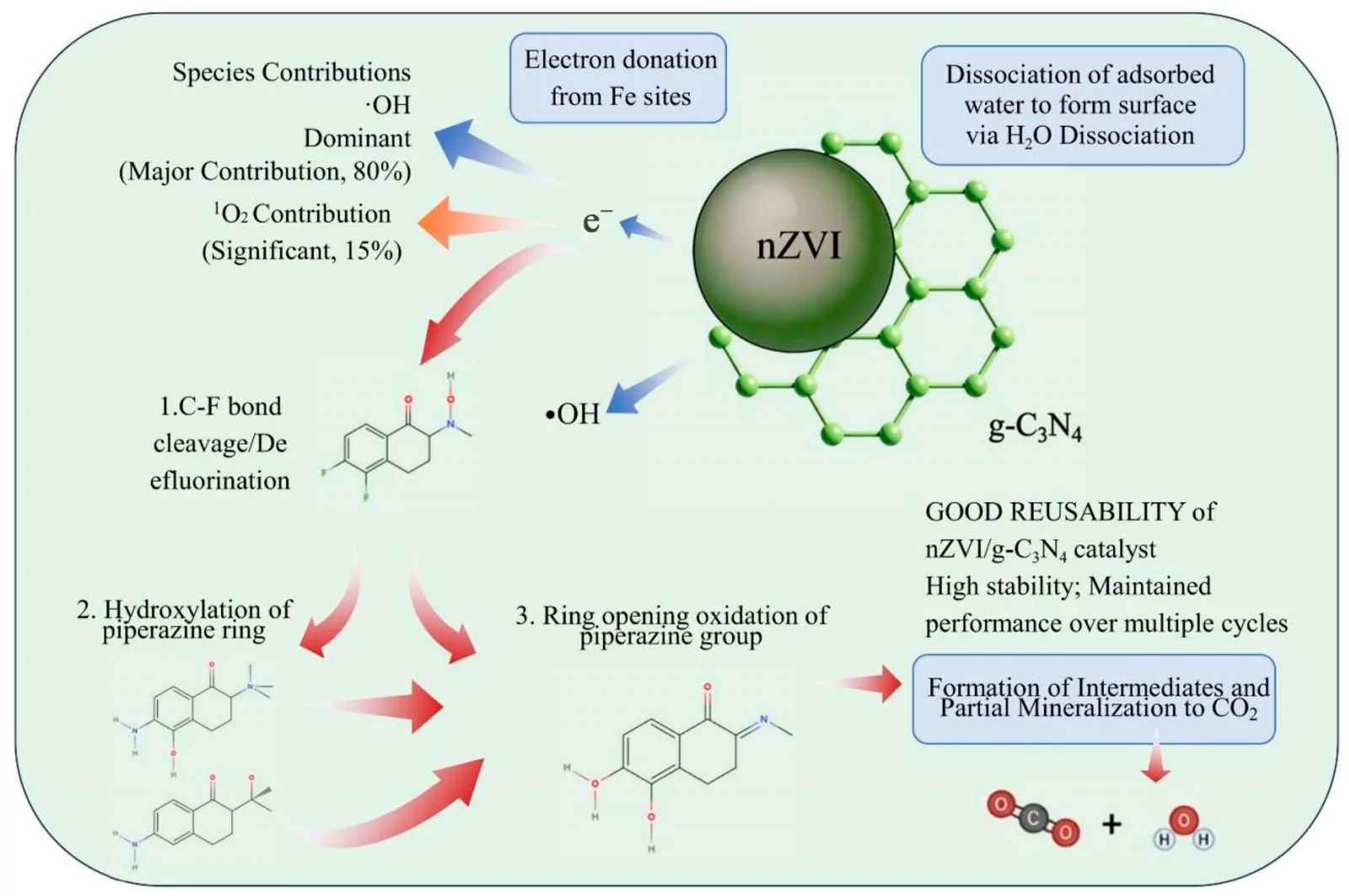
Pathways for fluoroquinolone removal in the nZVI/g-C_3_N_4_ system [55].

The oxides and hydroxides formed on the nZVI surface adsorb fluoroquinolone antibiotics through surface complexation [43,90]. Zhao et al. employed PEG-stabilized zeolite-supported nZVI (PZ-NZVI) for the removal of norfloxacin and ofloxacin, identifying adsorption as the primary removal mechanism, with the complexation reaction expressed as follows (see Figure 3) [43]:

Under anoxic conditions, nZVI attacks the C–F bond in fluoroquinolone molecules through electron transfer, resulting in defluorination (see Figure 3) [57,65]. Deng et al. detected F^−^ release in a DBD-nZVI/g-C_3_N_4_ system, confirming the defluorination process. Liu et al. observed that the concentration of F^−^ increased over time during norfloxacin degradation in an nZVI/peroxymonosulfate (PMS) system, indicating that defluorination is a sustained degradation pathway (see Equation (33)) [55,65].

(33) Fe-OH+HOOC-FQs→Fe-OOC-FQs+O

Under oxic conditions or in the presence of exogenous oxidants, nZVI activates dissolved oxygen or persulfate to generate reactive oxygen species (ROS), leading to the oxidative degradation of fluoroquinolone antibiotics [48,57,90]. Liu et al. investigated norfloxacin degradation in an nZVI/PMS system and confirmed through quenching experiments and EPR analysis that both SO_4_^⋅−^ and OH participate in the oxidative degradation, with SO_4_^⋅−^ being the dominant radical [65]. Comparison of methods for removing fluoroquinolone antibiotics using zero-valent iron nanoparticles is shown in Table 5.

## 4. Conclusions and Perspectives

NZVI (nZVI) exhibits promising potential for application in wastewater treatment owing to its strong reducing capability and high specific surface area. However, its practical application remains constrained by issues such as easy aggregation, rapid passivation, and poor electron selectivity [42,46,83]. Modification strategies including surface coating, support loading, metal doping, and sulfidation have been demonstrated to significantly enhance the dispersibility, stability, and reactivity of nZVI. Surface coating modification utilizes materials such as PEG, starch, chitosan, and aluminum hydroxide gel to encapsulate nZVI, inhibiting particle aggregation through steric hindrance and electrostatic repulsion while simultaneously shielding the particles from oxygen to delay passivation [3,7,61]. Support loading disperses nZVI onto porous materials such as activated carbon [2], biochar [49,95,96], zeolite [43,51], and graphene [55,76], which not only addresses the aggregation issue but also concentrates pollutants through adsorption, thereby shortening mass transfer distances. Sulfidation modification forms FeS and FeS_2_ layers on the nZVI surface, enhancing electron selectivity and expanding the applicable pH range [42,49,57]. In terms of stability, different modification strategies yield distinct performances. Surface coating with polymers such as starch or chitosan can effectively reduce iron leaching and maintain high reactivity over multiple cycles; for instance, starch-stabilized nZVI exhibited only a 6.22% efficiency loss after ten consecutive reuse cycles in a PS/US/nZVI system for flutamide degradation, with the removal efficiency declining from 97.2% to 91.0%, while the leached Fe^2+^ concentration progressively decreased from 12.4 mg/L in the first cycle to 7.1 mg/L in the tenth, indicating gradual passivation of surface active sites rather than structural collapse [44]. Sulfidation significantly improves electron selectivity and long-term stability by protecting the Fe^0^ core from rapid oxidation via the formation of conductive FeS_x_ layers; however, the reusability of S-nZVI remains moderate—in an ultrasound-mediated S-nZVI/peracetic acid system for enrofloxacin removal, the degradation efficiency declined from 91.86% in the first cycle to 74.35% and 62.88% in the second and third cycles, respectively, although the material retained good magnetic properties for recovery [49,57]. Metal doping or supporting nZVI on modified biochar can enhance catalytic lifetime by promoting the Fe^3+^/Fe^2+^ redox cycle and improving structural integrity; for example, MBC@nZVI (alkali-modified biochar-supported nZVI) maintained degradation efficiencies of 93.6%, 84.2%, and 79.1% over three consecutive cycles for naphthalene removal, and remarkably, it retained over 95% of its initial catalytic activity after 30 days of sealed storage, demonstrating that the biochar matrix effectively anchors nZVI particles and buffers the structural evolution caused by the Kirkendall effect (A solid-state diffusion phenomenon in which, at the interface between two different metals (or materials), atoms undergo unequal mutual diffusion due to differences in diffusion rates of the respective components. This leads to the formation of vacancies on the side with faster diffusion. These vacancies continuously accumulate and coalesce, eventually forming microvoids or even hollow structures.) during reaction [42]. Collectively, properly modified nZVI materials can meet the durability requirements for practical wastewater treatment, thereby strengthening their promise as scalable remediation technologies.

In terms of mechanism, the removal of antibiotics by nZVI primarily involves adsorption, reductive degradation, and oxidative degradation, with multiple mechanisms often acting synergistically in practical systems. The adsorption mechanism mainly encompasses electrostatic interactions, surface complexation, hydrogen bonding, and π-π interactions [43,51,76]. Under anoxic conditions, reductive degradation dominates, with nZVI reducing unsaturated functional groups in antibiotic molecules through direct electron transfer [97]. Under oxic conditions or in the presence of exogenous oxidants, oxidative degradation prevails, where nZVI activates dissolved oxygen, persulfate, or H_2_O_2_ to generate reactive oxygen species (ROS) [46,53].

Despite the significant progress achieved in the application of nZVI and its modified materials for antibiotic removal, several challenges remain to be addressed. First, nZVI readily undergoes side reactions with dissolved oxygen and water in aqueous solutions, leading to ineffective hydrogen evolution and reduced electron utilization efficiency [42]. Although sulfidation modification can mitigate this issue, the long-term stability of the sulfidation layer warrants further investigation [49,57]. In practical applications, the presence of natural organic matter and inorganic ions in water bodies competes for active sites and radicals, thereby affecting the degradation efficiency of Fenton-like systems [44,77]. Furthermore, current research predominantly focuses on the removal efficiency of specific antibiotics, with insufficient assessment and investigation of the ecotoxicity of degradation byproducts [77,98]. At present, the preparation of most modified nZVI materials remains at the laboratory stage, facing challenges such as high costs, complex processes, and difficulties in scale-up [42,83].

In conclusion, nZVI and its modified materials exhibit substantial potential in the field of antibiotic pollution control; however, the transition from laboratory research to engineering applications still faces numerous challenges. Nonetheless, through material innovation, mechanistic deepening, and technological integration, nZVI is expected to become one of the core technologies for antibiotic wastewater treatment in the future, making significant contributions to safeguarding aquatic ecosystem safety and human health [99,100,101].

## Figures and Tables

**Table 1:** Characteristics and environmental risks of typical antibiotic pollutants in medical wastewater [13,14].

Antibiotic Category	Representative Drug	Stability in Water	Main Sources	Compound Structural Formula
β-lactam classes	Amoxicillin, ceftriaxone, Imipenem	Half-life 1 to 7 days	Hospital antibiotic use, Pharmaceutical wastewater	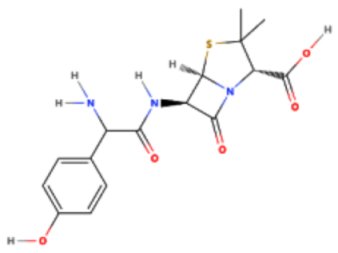 (Amoxicillin) 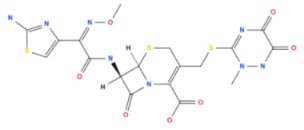 (Ceftriaxone) 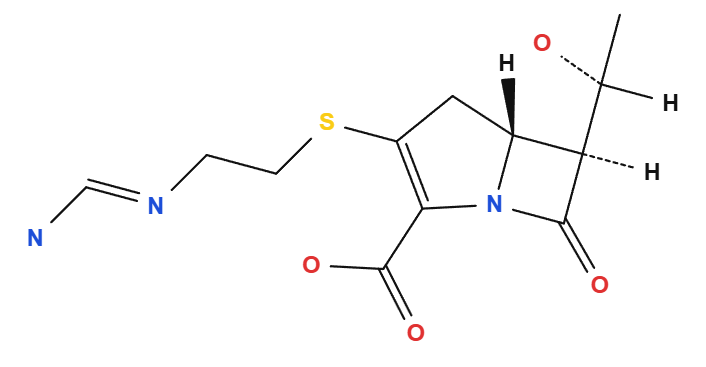 (Imipenem)
Quinolones	Ciprofloxacin, Enrofloxacin, Norfloxacin	Half-life 7 to 30 days	Clinical anti-infective treatment, Livestock farming	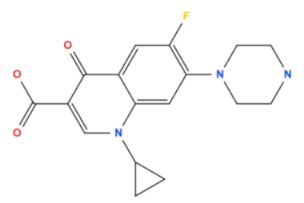 (Ciprofloxacin) 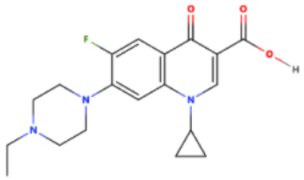 (Enrofloxacin) 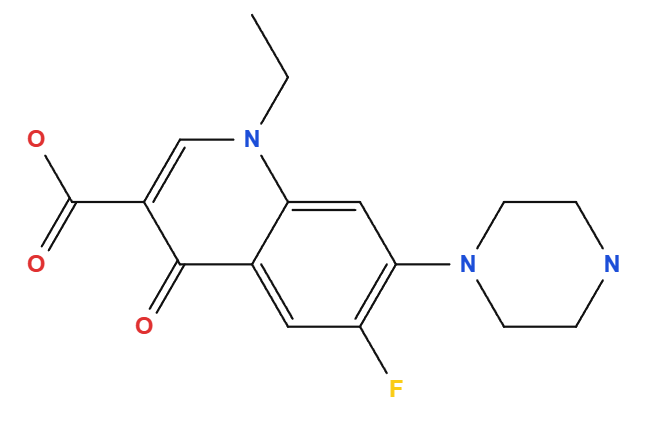 (Norfloxacin)
Macrolides	Roxithromycin, Erythromycin, Azithromycin	Half-life 3 to 10 days	Respiratory tract infection treatment, Veterinary antibiotics	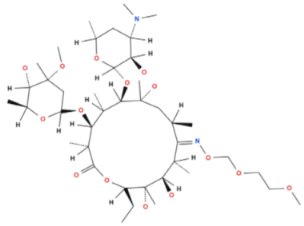 (Roxithromycin) 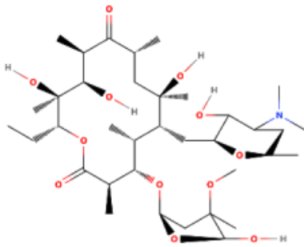 (Erythromycin) 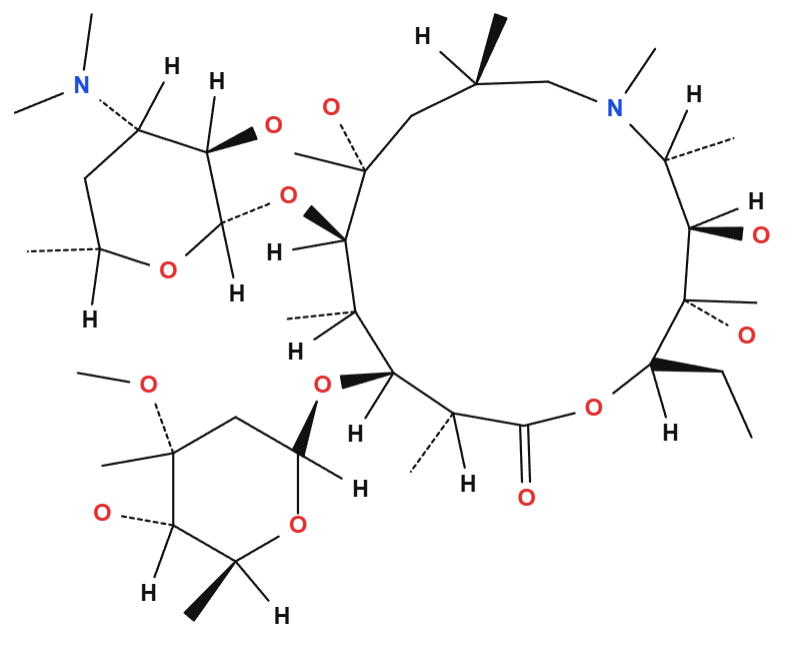 (Azithromycin)
Sulfonamides	Sulfamethoxazole, Sulfadiazine, Sulfapyridine	Half-life 10 to 20 days	Treatment for urinary tract infections, agricultural use	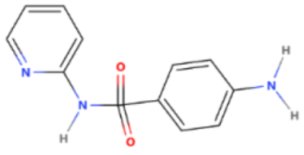 (Sulfapyridine) 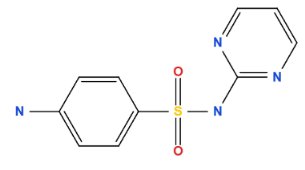 (Sulfadiazine) 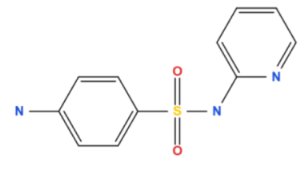 (Sulfapyridine)
Tetracyclines	Tetracycline, Chlortetracycline, Oxytetracycline	Half-life 5 to 15 days	Broad-spectrum antibacterial treatment, Aquaculture	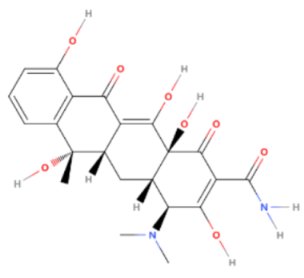 (Tetracycline) 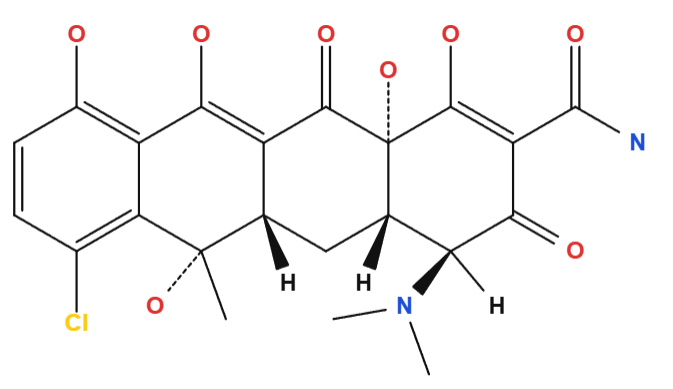 (Chlortetracycline) 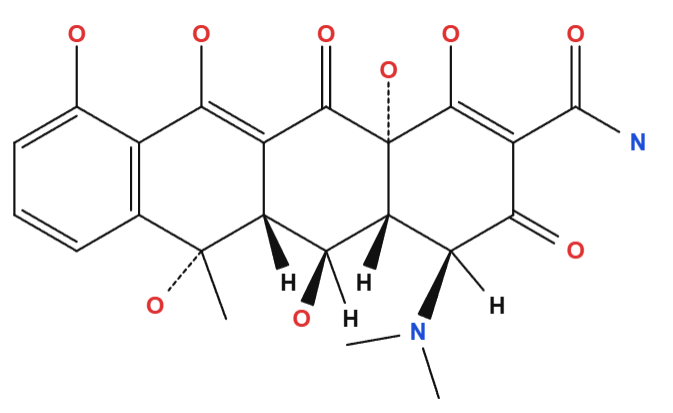 (Oxytetracycline)
Aminoglycosides	Gentamicin, Amikacin	Half-life: 1 to 3 days	Treatment for intestinal infections, Veterinary injectables	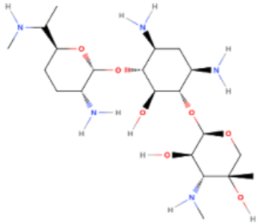 (Gentamicin) 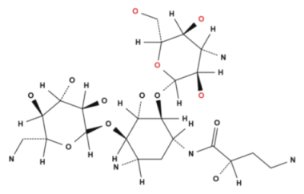 (Amikacin)

**Table 3:** Comparison of tetracycline removal performance by different nZVI-based materials.

Material Types	Modification Strategy	Reaction Conditions	Removal Efficiency	Main Mechanism	References
nZVI	Unmodified	[TC] = 30 mg/L, [nZVI] = 10 mg, 60 min, room temperature	48.86%	Adsorption + reduction	[51]
nZVI@AHG	Aluminum hydroxide gel coating	[TC] = 50 mg/L, [nZVI@AHG] = 0.5 g/L, 60 min, pH = 5–7	98.1%	Adsorption + flocculation + reduction	[29]
nZVI@ZSM	ZSM-5 molecular sieve loading	[TC] = 30 mg/L, [nZVI@ZSM] = 10 mg, 60 min, room temperature	84.00%	Adsorption + reduction	[51]
nZVI@C-ZSM	Chitosan-modified ZSM-5 loading	[TC] = 30 mg/L, [NZVI@C-ZSM] = 166.7 mg/L, 60 min, room temperature	97.72%	Adsorption + oxidation (·OH,·O^2−^)	[51]
nZVI@BC and hydroxyapatite	Co-doping of nZVI and HAP via pyrolysis-impregnation method	[TC] = 50 mg/L, [FPB-9] = 0.5 g/L, 48 h, pH = 6.5, 298 K	97.4%	synergistic adsorption+ heterogeneous Fenton degradation (·OH, H_2_O_2_)	[81]
porous carbon /nZVI composite	Spatial confinement via natural rice straw hierarchical channels and 2-methylimidazole (2-MI) coordination	[TC] = 40 mg/L, [MBC-2MI] = 0.1 g/L, [PMS] = 0.4 mM, 20 min, pH = 4, 25 °C	95.88%	PMS activation + oxidation (_1_O^2^)	[82]
ZnO/CFB	Carbon fiber supported ZnO (photocatalysis)	[OCA] = 30 mg/L, [ZnO/CFB] = 0.5 g/L, 120 min, pH = 8.0, 350 W Xe lamp	86.7%	Photocatalytic oxidation (·O^2−^)	[71]
BC-nZVI	Biochar loading	[TC] = 10 mg/L, [BC-nZVI] = 10 mg, [PS] = 0.25 mM, 120 min, pH = 3	99.57%	Adsorption + oxidation (·OH,·SO_4_^•−^)	[63]

**Table 4:** Comparison of the removal performance of β-lactam antibiotics by different nZVI materials.

Antibiotics	Material Type	Modification Strategies	Reaction Conditions	Removal Efficiency	Main Mechanism	Refer-ence
Amoxicillin (AMX)	nZVI	Impact of divalent cations (Cu^2+^, Mg^2+^) investigated,;No material modification involved	[AMX] = 40 mg/L, [nZVI] = 0.5 g/L, [H_2_O_2_] = 6.6 mM pH = 3, 50 min	90%	Heterogeneous Fenton-like oxidation; Cu^2+^ is reduced by Fe^0^ to form a Fe/Cu bimetal, accelerating Fe^2+^ generation and ·OH production; Mg^2+^ forms precipitates that inhibit the reaction.	[86]
AMX	B-nZVI/Ni	Bentonite support and nickel modification	[AMX] = 20 mg/L, Material dosage = 4 g/L, 60 min, pH = 7.3	94.6% (The combined removal efficiency of a mixed solution containing three antibiotics—amoxicillin (AMX), ampicillin (AMP), and penicillin (PCL)—within 60 min)	Adsorption and Catalytic Reductive Degradation	[54]
AMX	NZVI@GT-derived CDs	One-step hydrothermal synthesis of nZVI using the natural biopolymer gum tragacanth (GT)	[AMX] = 0.05 mM, [H_2_O_2_] = 20 mM, Catalyst dosage = 765 mg/L 60 min, pH = 3.5	90%	Heterogeneous Fenton-like reaction	[87]
Cephalexin (CEX)	PPAC-nZVI	Green synthesis of nanoparticles using pomegranate peel extract (PPE), combined with phosphoric acid (H_3_PO_4_) activated pomegranate peel activated carbon as a support material.	[CEX] = 50 mg/L, Material dosage = 1.2 g/L Adsorbent 0.4 g, 45 min, pH = 5	96.06%	Monolayer chemisorption and hydrogen bonding	[88]
CEX	NNZVI/TNZVI	Green synthesis utilizing Nettle and Thyme leaf extracts as reducing, capping, and stabilizing agents.	[CEX] = 25 mg/L, Material dosage = 0.1 g/L 120 min, pH = 2	Near 100%	Adsorption	[89]

**Table 5:** Comparison of the removal performance of Fluoroquinolone Antibiotic by different nZVI materials.

Antibiotics	Material Type	Modification Strategies	Reaction Conditions	Removal Efficiency	Main Mechanism	Refer-ence
Norfloxacin (NOR)	PZ-nZVI	PEG-4000 stabilization + zeolite loading	[NOR] = 10 mg/L, [PZ-nZVI] = 0.5 g/L, 60 min, pH = 6.5	98.42%	Surface complexation, hydrophobic interaction, pore filling, electrostatic interaction	[43]
NOR	S-nZVI	Sulfidation; CA/S-nZVI@Ps system with added citric acid (CA)	[NOR] = 50 mg/L, [S-nZVI] = 0.5 g/L 75 min, pH = 7	95.41%	Fenton-like oxidation (SO_4_^•−^,·OH)	[94]
NOR	nZVI	Unmodified	[NOR] = 25 mg/L, [nZVI] = 1 g/L, [PMS] = 0.8 mM, 60 min, pH = 7	92.0%	Fenton-like oxidation (SO_4_^•−^,·OH)	[65]
Ofloxacin (OFL)	PZ-nZVI	PEG-4000 stabilization + zeolite loading	[OFL] = 10 mg/L, [PZ-nZVI] = 0.4 g/L, 60 min, pH = 6.5	97.62%	Surface complexation, hydrophobic interaction, pore filling, electrostatic interaction	[43]
OFL	nZVI@PAC	Activated carbon loading	[OFL] = 25 mg/L, nZVI@PAC 125 mg/L, [H_2_O_2_] = 10 mM, 60 min, pH = 6	94.7%	Fenton-like oxidation (·OH, Fe(IV))	[48]
Ciprofloxacin (CIP)	nZVI/Cu-PVDF hybrid film	Immobilization in a PVDF polymer matrix via solvent casting	[CIP] = 10 ppm, [H_2_O_2_] = 30 μL/mL, sunlight irradiation (intensity: 95,000 lx, temp: 36 °C), 180 min, pH = 4	94%	Photocatalytic and photo-Fenton processes.	[15]
CIP	nZVI/PA	Polystyrene anion resin loading	[CIP] = 0.15 mM, DO, 360 min, pH = 5	98.5%	Adsorption + oxidation (·OH,·O^2−^)	[80]
CIP	nZVI/g-C_3_N_4_	g-C_3_N_4_ load	[CIP] = 10 mg/L, DBD plasma, 10 min, 12 kV	94.3%	Plasma oxidation (·OH)	[55]
CIP	nZVI@LDH	Coating with layered double hydroxide to form core–shell structure	1 V voltage, [nZVI@LDH] = 1 g/L, [CIP] = 5 mg/L, pH 6.8~7.5, HRT = 8 h	82%	Plasma oxidation (·OH)	[56]
Enrofloxacin (ENR)	S-nZVI	Vulcanization modification	[ENR] = 0.05 mM, [PAA] = 100 μM, US = 60 kHz, 10 min, pH = 7	91.86%	Contributions from ·OH, ·O_2_^−^, FeO^2+^, and RO· Fe-ENR complexation accelerates Fe^2+^ release	[57]
ENR	FeSBC-NS	N/S co-doping and Fe-C heterojunction construction	[FeSBC-NS] = 0.4 g/L, [PDS] = 3 mM, [ENR] = 20 mg/L, 30 min, pH 3.0~9.0	99.9%	synergistic electron transfer by heterojunction and N/S active sites	[84]

## Data Availability

No new data were created or analyzed in this study. Data sharing is not applicable to this article.
